# Rapid resolution of migraine symptoms after initiating the preventive treatment eptinezumab during a migraine attack: results from the randomized RELIEF trial

**DOI:** 10.1186/s12883-022-02714-1

**Published:** 2022-06-03

**Authors:** Jessica Ailani, Peter McAllister, Paul K. Winner, George Chakhava, Mette Krog Josiassen, Annika Lindsten, Bjørn Sperling, Anders Ettrup, Roger Cady

**Affiliations:** 1 Department of Neurology, Georgetown University Hospital, Washington, DC USA; 2 New England Institute for Neurology and Headache, Stamford, CT USA; 3 Palm Beach Headache Center, West Palm Beach, FL USA; 4 Georgian Association of Medical Specialties, Multiprofile Clinic Consilium Medulla, D.Tvildiani Medical University, Tbilisi, Georgia; 5 H. Lundbeck A/S, Copenhagen, Denmark; 6 Lundbeck LLC, Deerfield, IL USA; 7 RK Consults, Ozark, MO USA

**Keywords:** Eptinezumab, Migraine, CGRP, MBS

## Abstract

**Background:**

Eptinezumab is an anti-calcitonin gene-related peptide (CGRP) monoclonal antibody approved for the preventive treatment of migraine. In the phase 3 RELIEF study, eptinezumab resulted in shorter time to headache pain freedom and time to absence of most bothersome symptom (MBS; including nausea, photophobia, or phonophobia) compared with placebo when administered during a migraine attack. The objective of this exploratory analysis was to examine the earliest time points that eptinezumab separated from placebo (*P* < .05) on headache- and migraine-associated symptoms when administered during a migraine attack.

**Methods:**

RELIEF, a multicenter, parallel-group, double-blind trial, occurred from November 7, 2019, through July 8, 2020. Adults considered candidates for preventive treatment were randomized to eptinezumab 100 mg (*N* = 238) or placebo (*N* = 242) administered intravenously over 30 min within 1–6 h of migraine onset. Outcome measures included headache pain freedom/relief and absence of MBS, patient’s choice of photophobia, phonophobia, or nausea, at regular intervals from 0.5 to 48 h after infusion start. Censoring was applied at time of acute rescue medication use.

**Results:**

At hour 1, more eptinezumab-treated patients achieved headache pain freedom (9.7%), headache pain relief (38.7%), and absence of MBS (33.2%) versus placebo (4.1%, 26.9%, and 22.1%, respectively; *P* < .05 all), with separation from placebo (*P* < .05) through hour 48. Eptinezumab separated from placebo (*P* < .05) at hour 1 in absence-of-photophobia (29.4% vs 17.0%) and absence-of-phonophobia (41.2% vs 27.2%) and through hour 48. Initial separation from placebo (*P* < .05) in absence-of-nausea occurred at end-of-infusion (0.5 h; 36.7% vs 25.4%, respectively).

**Conclusion:**

Preventive treatment with eptinezumab initiated during a migraine attack resulted in more patients achieving headache pain freedom/relief and absence of MBS, with separation from placebo (*P* < .05) as early as 0.5–1 h following the start of infusion. Rapid resolution of headache- and migraine-associated symptoms by a peripherally acting, intravenously administered antibody suggest a peripheral site of pharmacological action for CGRP blockade.

**Trial registration:**

ClinicalTrials.gov Identifier: NCT04152083.

**Supplementary Information:**

The online version contains supplementary material available at 10.1186/s12883-022-02714-1.

## Background

Migraine has been shown to be among the most disabling disorders worldwide, and it continues to be a major deterrent of health [1]. The age group with the highest burden of disease (as estimated by years lived with a disability) is 15**–**49 years, representing a period when family, education, and career building are of top priority [1]. Despite receiving preventive treatment, patients may still require acute migraine medication because of delayed onset of effect of the preventive medication, or because the preventive treatment failed to adequately prevent the bothersome symptoms of migraine. Approximately 65% of patients with migraine have reported experiencing all three cardinal-associated symptoms of migraine: nausea, photophobia, and phonophobia, which comprise the options for patient selection of most bothersome symptom (MBS) [2]. In addition to headache pain freedom, efficacy trials often use MBS freedom as a co-primary or key secondary efficacy endpoint [2].

Eptinezumab, a humanized IgG1 monoclonal antibody that binds rapidly, durably, and with high affinity to calcitonin gene-related peptide (CGRP) [3], is approved for the preventive treatment of migraine in adults [4]. Eptinezumab provides sustained blockade of this key neuropeptide’s interaction with its receptor [3] and, having a molecular weight of 140 KD, is believed not to cross the blood–brain barrier [5], yet has demonstrated rapid and sustained efficacy in preventing migraine [6–9]. This effect may aid in elucidating peripheral versus central migraine mechanisms important to understanding the pathophysiology of migraine as well as the pharmacological site of action for treatment approaches that inhibit CGRP signaling [10].

RELIEF, a phase 3, multicenter, double-blind trial in patients experiencing migraine 4–15 days/month, found that eptinezumab, when administered during a migraine attack, demonstrated benefit by reducing headache, migraine-associated symptoms, and rescue medication use compared with placebo [7]. At 2 h after infusion start, more eptinezumab-treated patients achieved headache pain freedom and absence of MBS compared with those receiving placebo. Furthermore, eptinezumab delayed time to next migraine, as evidenced by a median time to next migraine of 10 days in the eptinezumab group versus 5 days in the placebo group (*P* < 0.001) [7].

The objective of this exploratory analysis of the RELIEF study was to evaluate if separation from placebo (*P* < 0.05) was achieved at time of first assessment (0.5 h) or the earliest assessment thereafter in headache pain freedom, headache pain relief, absence of MBS, and absence of photophobia, phonophobia, and nausea individually.

## Methods

### Study design, procedures, and patients

Detailed methodology for RELIEF (NCT04152083) has been published [7]. Briefly, RELIEF was a 4- to 12-week, phase 3, multicenter, parallel-group, double-blind, placebo-controlled clinical trial conducted between November 2019 and July 2020 in which patients were randomized to receive eptinezumab 100 mg or placebo intravenously during a moderate to severe migraine attack. Patients were age 18–75 years with a > 1-year history of migraine (defined by the International Classification of Headache Disorders, 3^rd^ edition criteria) [11], with or without aura, with onset of first migraine before 50 years of age, and experiencing migraine on 4–15 days per month in the 3 months prior to screening. Additionally, patients were required to have a history of migraine attacks with a duration of 4–72 h untreated, headache pain of moderate to severe intensity, and a patient-selected MBS from among nausea, photophobia, or phonophobia. Patients were also required to have a history of either previous or active use of triptans for migraine. These criteria were utilized to ensure that patients who could be candidates for preventive treatment were enrolled and to allow patients with more severe chronic migraine to enroll (a deviation from current guidance [12, 13] for studies of acute migraine treatment that limits the population to 2–8 monthly migraine days).

Treatment (total volume of 100 mL) was administered intravenously over a period of 30 min on day 0 within 1–6 h of onset of the qualifying migraine with moderate to severe headache. Rescue medication (any acute medication to treat migraine or migraine-associated symptoms) was not permitted in the 24-h period prior to receiving study treatment or within 2 h of infusion start. After 2 h following infusion start, patients were permitted to take rescue medication if they continued to experience moderate or severe headache pain or had significant migraine-associated symptoms. Additionally, if initial migraine relief was achieved at 2 h, but headache- or migraine-associated symptoms returned within 2‒48 h of study drug administration, rescue medication was permitted. Rescue medication taken prior to 2 h post infusion start was considered a protocol deviation, but all patients were included in the analysis and censored at the time of use (< 5 patients per treatment arm within the first 2 h).

### Outcome measures

The number of patients achieving headache pain freedom and headache pain relief was recorded at 0.5, 1, 1.5, 2.5, 3, 3.5, 4, 6, 9, 12, 24, and 48 h after infusion start. Headache pain was rated on a 4-point scale where 3 = severe, 2 = moderate, 1 = mild, and 0 = no pain. Pain was required to be 2 or 3 at baseline, with headache pain freedom defined as no pain (0) and headache pain relief defined as mild or no pain (0 or 1). Additionally, the number of patients who experienced absence of patient-selected MBS and absence of photophobia, phonophobia, and nausea individually was recorded at each time point. The individual analyses of absence of photophobia, phonophobia, and nausea were conducted in patients who experienced that symptom at baseline, whether or not they selected this symptom as their MBS.

Use of rescue medication was recorded at each time point. The time to headache pain freedom/absence of MBS was censored at the time of first rescue medication if used prior to obtaining pain freedom/absence of MBS. Thus, for patients who reported pain freedom/absence of MBS without using rescue medication up to and including the time point where pain freedom/absence of MBS was reported, the first collected time point with pain freedom was included in the analysis (pain freedom obtained). For patients who reported taking rescue medication without having reported pain freedom/absence of MBS at an earlier time point, censoring was applied at the first time point where rescue medication was used. Finally, if a patient did not report pain freedom/absence of MBS during the 48 h and did not report taking rescue medication at any time point, censoring was applied at the time point of the last recorded observation during the 48 h.

### Statistical analyses

The percentage of patients with headache pain freedom, absence of MBS at 2 and 4 h, and rescue medication use within 24 h post infusion were secondary efficacy endpoints, with multiplicity control of the type 1 error. Headache pain freedom, headache pain relief, absence of MBS, and absence of photophobia, phonophobia, and nausea at all other specified time points were exploratory efficacy endpoints. Headache pain freedom/relief and absence of MBS were analyzed in all patients; absence of photophobia, phonophobia, and nausea was analyzed in patients experiencing the corresponding symptom with their qualifying migraine. If an assessment was missing at a given time point, it was assumed that no freedom/relief or absence was achieved, and no rescue medication was used at that time point.

All measures were assessed descriptively or tested at a 2-sided 5% alpha with no adjustment for multiplicity. Each of these measures were compared between the eptinezumab and placebo groups at each of the specified time points using a Cochran–Mantel–Haenszel test, stratified for concomitant migraine preventive treatment use and region. The Mantel–Haenszel estimates of treatment difference, odds ratio, and associated confidence intervals were calculated for each comparison. Sensitivity analyses of all endpoints were conducted where use of rescue medication was allowed, that is, without applying censoring of data.

## Results

### Patients

A total of 238 and 242 patients were randomized to and received eptinezumab 100 mg and placebo, respectively. Baseline demographics and characteristics have been reported previously [7] and showed similarity between eptinezumab and placebo groups. Briefly, patients had an average of 7 monthly migraine days at baseline, the average duration of the qualifying migraine before the start of infusion was 3.7 h, and there was a nearly 50–50 split in moderate (47.3%) versus severe (52.3%) headache pain intensity. Nearly all patients (96.7%) had presence of photophobia with their qualifying migraine, followed by phonophobia in 82.3% and nausea in 72.7% of patients. A total of 228/480 (47.5%), 157/480 (32.7%), and 93/480 (19.4%) patients identified photophobia, nausea, or phonophobia, respectively, as their MBS. Two patients receiving placebo did not have an MBS reported at baseline due to technical issues with the eDiary. Among patients who experienced all three symptoms (photophobia, phonophobia, *and* nausea) with their qualifying migraine (*n* = 278), the most selected MBS was nausea (*n* = 120, 43.2%), followed by photophobia (*n* = 105, 37.8%) and phonophobia (*n* = 53, 19.1%).

### Time course to headache pain freedom, headache pain relief, and absence of MBS

**Fig. 1:**
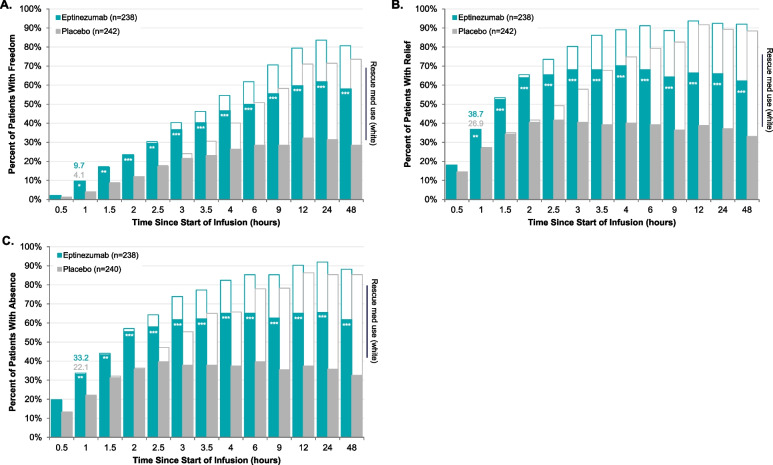
Time Course to Headache Pain Freedom (**A**), Headache Pain Relief (**B**), and Absence of MBS (**C**). *P < 0.05, **P <0 .01, ***P < 0.001 vs placebo in analysis censoring for use of rescue medication. The teal/gray bars represent the percentage of patients achieving headache pain freedom (**A**), headache pain relief (**B**), and absence of MBS (**C**) without rescue medication use prior to the achievement. The white bars represent the percent of patients achieving headache pain freedom (**A**), headache pain relief (**B**), and absence of MBS (**C**) regardless of rescue medication use

The percentage of patients achieving headache pain freedom, headache pain relief, and absence of MBS at each time point, with and without censoring for use of rescue medication, are detailed in Supplemental Table 1. Censoring for use of rescue medication, eptinezumab separation from placebo for headache pain freedom was observed from hour 1 after infusion start through hour 48 (*P* < 0.05, all time points; Fig. 1A). Similar results were observed for headache pain relief (Fig. 1B) and absence of patient-selected MBS (Fig. 1C), with eptinezumab separating from placebo from hour 1 through hour 48 (*P* < 0.05, all time points).

### Time course to absence of photophobia, phonophobia, and nausea

**Fig. 2:**
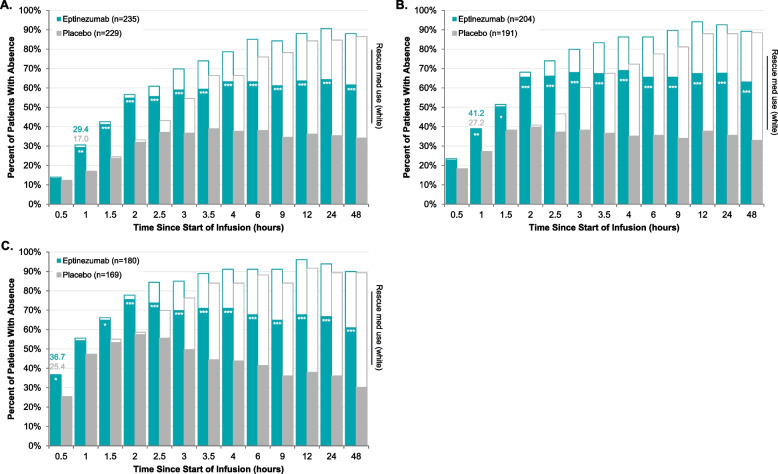
Time Course to Absence of Photophobia (**A**), Phonophobia (**B**), and Nausea (**C**). *P < 0.05, **P < 0.01, ***P < 0.001 vs placebo in analysis censoring for use of rescue medication. Analyses were conducted in patients experiencing the corresponding symptom with their qualifying migraine. The teal/gray bars represent the percentage of patients achieving absence of photophobia (**A**), phonophobia (**B**), and nausea (**C**) without rescue medication use prior to the achievement. The white bars represent the percent of patients achieving absence of photophobia (**A**), phonophobia (**B**), and nausea (**C**) regardless of rescue medication use

The percentage of patients achieving absence of photophobia, absence of phonophobia, and absence of nausea at each time point, with and without censoring for use of rescue medication, are detailed in Supplemental Table 2. Censoring for use of rescue medication, in patients experiencing photophobia with their qualifying migraine, eptinezumab separated from placebo (*P* < 0.05) in absence of photophobia from hour 1 through hour 48 (Fig. 2A). Similarly, eptinezumab separation from placebo (*P* < 0.05) was noted from hour 1 through hour 48 for absence of phonophobia in patients experiencing phonophobia with their qualifying migraine (Fig. 2B). For absence of nausea, separation from placebo (*P* < 0.05) was observed at 0.5 h after infusion start in patients experiencing nausea with their qualifying migraine, and while separation from placebo was not statistically significant at the 1-h time point, it was achieved from the 1.5-h time point through 48 h (Fig. 2C).

### Use of rescue medication during the first 48 h after infusion

**Fig. 3:**
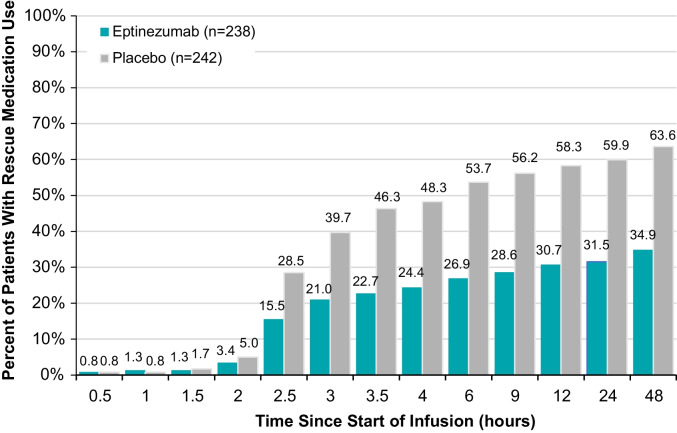
Cumulative Percent of Patients Using Rescue Medication After Start of Infusion. Patients were counted at the first time point of rescue medication use

Over the 48 h after the start of infusion, a larger percentage of patients receiving placebo required rescue medication as compared with patients receiving eptinezumab (Fig. 3). Within the first 48 h, 63.6% of patients receiving placebo had at some time point taken rescue medication compared with 34.9% of patients receiving eptinezumab.

## Discussion

The results of the present analyses showed that initiation of treatment with eptinezumab during an active migraine—in patients eligible for preventive migraine treatment—provided faster resolution in migraine associated symptoms (nausea, photophobia, and phonophobia) compared to treatment with placebo. Absence of nausea—the symptom most often identified as MBS by patients experiencing nausea, photophobia, and phonophobia with their qualifying migraine—separated from placebo (*P* < 0.05) observed at first time of assessment at 0.5 h following the start of the 30-min infusion (i.e., at the end of infusion), while absence of photophobia and phonophobia separated from placebo as early as 1 h (i.e., 0.5 h after the end of infusion). Treatment with eptinezumab also provided faster achievement of headache pain freedom, headache pain relief, and absence of MBS, with separation from placebo (*P* < 0.05) as early as 1 h following the start of the 30-min infusion. Importantly, eptinezumab decreased the need for rescue medication compared to placebo, but even when used, faster resolution or relief was still noted in eptinezumab-treated patients. Separation from placebo (*P* < 0.05) occurred until at least hour 24 for headache pain freedom, hour 6 for headache pain relief, and hour 9 for absence of MBS. The sustained efficacy through hour 48 indicates minimal breakthrough headache. These results are critical because preventive medications have historically taken several weeks to become efficacious.

The International Headache Society recommends headache pain freedom at 2 h as the primary efficacy parameter, with pain relief at 2 h as a secondary endpoint [13]; accordingly, most trials are designed as such when evaluating therapy intended for acute migraine treatment. The results of the present analyses found that eptinezumab onset of effect as observed by the earliest time point of separation from placebo was at 1 h for both headache pain freedom and headache pain relief. Furthermore, eptinezumab therapeutic gain (eptinezumab minus placebo) was 13% at 1 h and increased steadily to 38% at 3 h for headache pain freedom. By comparison, although not a head-to-head trial, the therapeutic gain for headache pain freedom of ubrogepant in the double-blind, single-attack, phase 3 ACHIEVE-1 and ACHIEVE-II trials was -1% at 1 h rising to 19% at 4 h [14–16], and the therapeutic gain for headache pain freedom of rimegepant in the double-blind, single-attack, phase 3 clinical trial was 7.6% at 2 h [17].

Administration of eptinezumab during an acute attack of migraine resolves migraine symptomatology quickly and may allow patients to return to their normal activities sooner. Although the mechanism of action is not completely understood, eptinezumab binds to CGRP, thus disrupting CGRP-induced trigeminal nociceptive transmission; in turn this improves acute migraine symptoms, and, over time, decreases migraine frequency [10]. Of note is the rapid achievement of absence of nausea in eptinezumab-treated patients compared with those who received placebo (separated from placebo at 0.5 h after the start of the 30-min infusion). Under physiological conditions, eptinezumab is unlikely to cross the blood‒brain barrier [5]. As such, eptinezumab’s site of pharmacologic action seemingly is in the periphery—where CGRP levels have been shown to be elevated during migraine attacks [5]. However, previous studies have concluded that nausea is centrally driven in patients with migraine [18]. However, since the results demonstrated rapid resolution of headache- and migraine-associated symptoms by a peripherally acting antibody this suggests that the therapeutic effect of treatments blocking CGRP signalling act primarily through peripheral mechanisms. Thus, these data also point to a critical role of the peripheral blockade of CGRP in the physiology of migraine-associated symptom resolution, even if such symptoms may arise by central mechanisms.

### Limitations

There are several limitations to consider, including that this study includes exploratory endpoints that were preplanned and consists of other time points than those used for the secondary analyses. Further, the RELIEF study was done in a clinical trial setting limiting the overall generalizability of these results. Furthermore, patients reported a higher migraine severity than previously reported in similar acute migraine trial populations, which may limit the comparison to other acute trials.

## Conclusion

Compared to placebo, eptinezumab demonstrated faster achievement of headache pain freedom, headache pain relief, and MBS freedom, as well as reduced rescue medication use. Additionally, the rapid improvement in migraine-associated symptoms, in particular nausea, separating from placebo earlier than pain relief or freedom, suggest an important role of the peripheral blockade of CGRP in the resolution of nausea independent of pain relief, and in resolving acute migraine symptomatology.

## Supplementary Information

**Additional file 1: Table S1.** Time After Start of Infusion to Headache Pain Freedom, Headache Pain Relief, and Absence of MBS. **Table S2.** Time After Start of Infusion to Absence of Photophobia, Phonophobia, and Nausea.

## Data Availability

In accordance with EFPIA’s and PhRMA’s “Principles for Responsible Clinical Trial Data Sharing” guidelines, Lundbeck is committed to responsible sharing of clinical trial data in a manner that is consistent with safeguarding the privacy of patients, respecting the integrity of national regulatory systems, and protecting the intellectual property of the sponsor. The protection of intellectual property ensures continued research and innovation in the pharmaceutical industry. Deidentified data are available to those whose request has been reviewed and approved through an application submitted to https://www.lundbeck.com/global/our-science/clinical-data-sharing.

## Abbreviations

CGRP: Calcitonin gene-related peptide
MBS: Most bothersome symptom
