# Correction: Rapid resolution of migraine symptoms after initiating the preventive treatment eptinezumab during a migraine attack: results from the randomized RELIEF trial

**DOI:** 10.1186/s12883-023-03368-3

**Published:** 2023-09-06

**Authors:** Jessica Ailani, Peter McAllister, Paul K. Winner, George Chakhava, Mette Krog Josiassen, Annika Lindsten, Bjørn Sperling, Anders Ettrup, Roger Cady

**Affiliations:** 1 Department of Neurology, Georgetown University Hospital, Washington, DC USA; 2 New England Institute for Neurology and Headache, Stamford, CT USA; 3 Palm Beach Headache Center, West Palm Beach, FL USA; 4 Georgian Association of Medical Specialties, Multiprofile Clinic Consilium Medulla, D.Tvildiani Medical University, Tbilisi, Georgia; 5 H. Lundbeck A/S, Copenhagen, Denmark; 6 Lundbeck LLC, Deerfield, IL USA; 7 RK Consults, Ozark, MO USA

**Correction: BMC Neurol 22, 205 (2022)**

**https://doi.org/10.1186/s12883-022-02714-1**

Following﻿ publication of the original article [1], the authors identified an error in Figs. 1 and 2. The correct figures are given below.

**Fig. 1:**
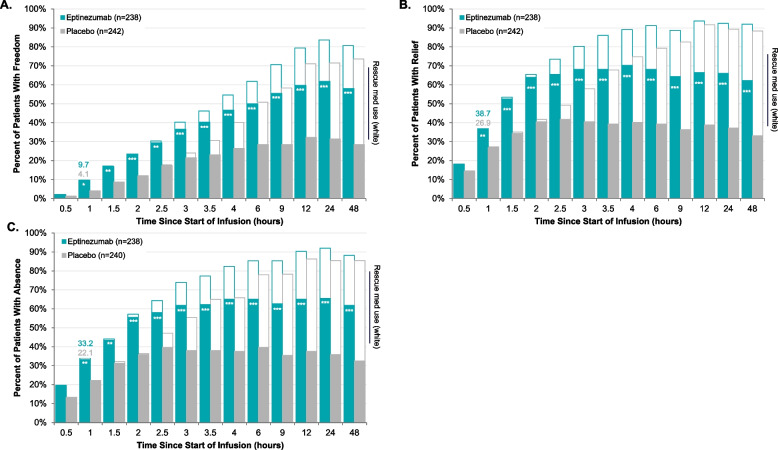
Time Course to Headache Pain Freedom (**A**), Headache Pain Relief (**B**), and Absence of MBS (**C**). *P < 0.05, **P < 0 0.01, ***P < 0.001 vs placebo in analysis censoring for use of rescue medication. The teal/gray bars represent the percentage of patients achieving headache pain freedom (**A**), headache pain relief (**B**), and absence of MBS (**C**) without rescue medication use prior to the achievement. The white bars represent the percent of patients achieving headache pain freedom (**A**), headache pain relief (**B**), and absence of MBS (**C**) regardless of rescue medication use

**Fig. 2:**
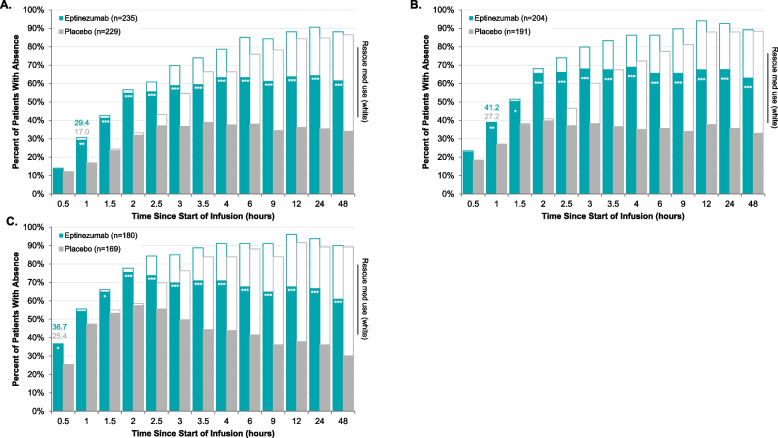
Time Course to Absence of Photophobia (**A**), Phonophobia (**B**), and Nausea (**C**). *P < 0.05, **P < 0.01, ***P < 0.001 vs placebo in analysis censoring for use of rescue medication. Analyses were conducted in patients experiencing the corresponding symptom with their qualifying migraine. The teal/gray bars represent the percentage of patients achieving absence of photophobia (**A**), phonophobia (**B**), and nausea (**C**) without rescue medication use prior to the achievement. The white bars represent the percent of patients achieving absence of photophobia (**A**), phonophobia (**B**), and nausea (**C**) regardless of rescue medication use

The original article [1] has been updated.
