# Supplementary material for: Rapid resolution of migraine symptoms after initiating the preventive treatment eptinezumab during a migraine attack: results from the randomized RELIEF trial
Source: BMC Neurol. 2022 Jun 3;22:205. doi: 10.1186/s12883-022-02714-1 (PMC9164335; doi:10.1186/s12883-022-02714-1)
Supplement: Supplementary file 1 — Additional file 1: Table S1. Time After Start of Infusion to Headache Pain Freedom, Headache Pain Relief, and Absence of MBS. Table S2. Time After Start of Infusion to Absence of Photophobia, Phonophobia, and Nausea. [file 12883_2022_2714_MOESM1_ESM.docx]

**Supplemental Table 1. Time After Start of Infusion to Headache Pain Freedom, Headache Pain Relief, and Absence of MBS**

|  | **With Rescue Medication Censoring** | | | | | | | | |
| --- | --- | --- | --- | --- | --- | --- | --- | --- | --- |
|  | **Headache Pain Freedom** | | | **Headache Pain Relief** | | | **Absence of MBS** | | |
| *Patients, n (%)* | **Eptinezumab 100 mg (n=238)** | **Placebo (n=242)** | ***P*-value** | **Eptinezumab 100 mg (n=238)** | **Placebo (n=242)** | ***P*-value** | **Eptinezumab**  **100 mg (n=238)** | **Placebo (n=240)** | ***P*-value** |
| **0.5 hours** | 5 (2.1) | 3 (1.2) | .46 | 43 (18.1) | 35 (14.5) | .27 | 46 (19.3) | 32 (13.3) | .08 |
| **1 hour** | 23 (9.7) | 10 (4.1) | .0162 | 92 (38.7) | 65 (26.9) | .0050 | 79 (33.2) | 53 (22.1) | .0067 |
| **1.5 hours** | 40 (16.8) | 21 (8.7) | .0073 | 125 (52.5) | 83 (34.3) | <.0001 | 103 (43.3) | 75 (31.3) | .0063 |
| **2 hours** | 56 (23.5) | 29 (12.0) | .0009 | 152 (63.9) | 98 (40.5) | <.0001 | 132 (55.5) | 86 (35.8) | <.0001 |
| **2.5 hours** | 70 (29.4) | 42 (17.4) | .0018 | 156 (65.5) | 101 (41.7) | <.0001 | 138 (58.0) | 95 (39.6) | <.0001 |
| **3 hours** | 87 (36.6) | 52 (21.5) | .0003 | 162 (68.1) | 98 (40.5) | <.0001 | 147 (61.8) | 91 (37.9) | <.0001 |
| **3.5 hours** | 90 (40.3) | 56 (23.1) | <.0001 | 162 (68.1) | 95 (39.3) | <.0001 | 148 (62.2) | 91 (37.9) | <.0001 |
| **4 hours** | 111 (46.6) | 64 (26.4) | <.0001 | 167 (70.2) | 97 (40.1) | <.0001 | 155 (65.1) | 90 (37.5) | <.0001 |
| **6 hours** | 119 (50.0) | 69 (28.5) | <.0001 | 162 (68.1) | 95 (39.3) | <.0001 | 155 (65.1) | 95 (39.6) | <.0001 |
| **9 hours** | 132 (55.5) | 69 (28.5) | <.0001 | 153 (64.3) | 88 (36.4) | <.0001 | 149 (62.6) | 85 (35.4) | <.0001 |
| **12 hours** | 142 (59.7) | 78 (32.2) | <.0001 | 158 (66.4) | 94 (38.8) | <.0001 | 155 (65.1) | 90 (37.5) | <.0001 |
| **24 hours** | 147 (61.8) | 76 (31.4) | <.0001 | 157 (66.0) | 90 (37.2) | <.0001 | 156 (65.5) | 86 (35.8) | <.0001 |
| **48 hours** | 138 (58.0) | 69 (28.5) | <.0001 | 148 (62.2) | 80 (33.1) | <.0001 | 147 (61.8) | 78 (32.5) | <.0001 |
|  | **Without Rescue Medication Censoring** | | | | | | | | |
|  | **Headache Pain Freedom** | | | **Headache Pain Relief** | | | **Absence of MBS** | | |
| *Patients, n (%)* | **Eptinezumab 100 mg (n=238)** | **Placebo (n=242)** | ***P*-value** | **Eptinezumab 100 mg (n=238)** | **Placebo (n=242)** | ***P*-value** | **Eptinezumab**  **100 mg (n=238)** | **Placebo (n=240)** | ***P*-value** |
| **0.5 hours** | 5 (2.1) | 3 (1.2) | .46 | 43 (18.1) | 35 (14.5) | .27 | 47 (19.7) | 32 (13.3) | .06 |
| **1 hour** | 23 (9.7) | 10 (4.1) | .0162 | 95 (39.9) | 66 (27.3) | .0027 | 81 (34.0) | 53 (22.1) | .0037 |
| **1.5 hours** | 41 (17.2) | 21 (8.7) | .0051 | 127 (53.4) | 85 (35.1) | <.0001 | 105 (44.1) | 77 (32.1) | .0066 |
| **2 hours** | 56 (23.5) | 29 (12.0) | .0009 | 156 (65.5) | 101 (41.7) | <.0001 | 136 (57.1) | 87 (36.3) | <.0001 |
| **2.5 hours** | 72 (30.3) | 43 (17.8) | .0013 | 175 (73.5) | 119 (49.2) | <.0001 | 153 (64.3) | 113 (47.1) | .0002 |
| **3 hours** | 96 (40.3) | 58 (24.0) | .0001 | 191 (80.3) | 140 (57.9) | <.0001 | 176 (73.9) | 133 (55.4) | <.0001 |
| **3.5 hours** | 110 (46.2) | 74 (30.6) | .0004 | 205 (86.1) | 164 (67.8) | <.0001 | 184 (77.3) | 156 (65.0) | .0028 |
| **4 hours** | 130 (54.6) | 97 (40.1) | .0014 | 212 (89.1) | 181 (74.8) | <.0001 | 196 (82.4) | 158 (65.8) | <.0001 |
| **6 hours** | 147 (61.8) | 123 (50.8) | .0160 | 217 (91.2) | 192 (79.3) | .0003 | 203 (85.3) | 187 (77.9) | .0373 |
| **9 hours** | 168 (70.6) | 141 (58.3) | .0047 | 211 (88.7) | 200 (82.6) | .06 | 203 (85.3) | 188 (78.3) | .0494 |
| **12 hours** | 189 (79.4) | 172 (71.1) | .0335 | 223 (93.7) | 222 (91.7) | .41 | 215 (90.3) | 207 (86.3) | .17 |
| **24 hours** | 199 (83.6) | 173 (71.5) | .0014 | 220 (92.4) | 216 (89.3) | .23 | 219 (92.0) | 205 (85.4) | .0234 |
| **48 hours** | 192 (80.7) | 178 (73.6) | .06 | 219 (92.0) | 214 (88.4) | .19 | 210 (88.2) | 205 (85.4) | .37 |

MBS, most bothersome symptom.

**Supplemental Table 2. Time After Start of Infusion to Absence of Photophobia, Phonophobia, and Nausea**

|  | **With Rescue Medication Censoring** | | | | | | | | |
| --- | --- | --- | --- | --- | --- | --- | --- | --- | --- |
|  | **Absence of Photophobia** | | | **Absence of Phonophobia** | | | **Absence of Nausea** | | |
| *Patients, n (%)* | **Eptinezumab**  **100 mg (n=235)** | **Placebo (n=229)** | ***P*-value** | **Eptinezumab**  **100 mg (n=204)** | **Placebo (n=191)** | ***P*-value** | **Eptinezumab**  **100 mg (n=180)** | **Placebo (n=169)** | ***P*-value** |
| **0.5 hours** | 32 (13.6) | 28 (12.2) | .67 | 47 (23.0) | 35 (18.3) | .27 | 66 (36.7) | 43 (25.4) | .0226 |
| **1 hour** | 69 (29.4) | 39 (17.0) | .0017 | 84 (41.2) | 52 (27.2) | .0041 | 98 (54.4) | 80 (47.3) | .16 |
| **1.5 hours** | 97 (41.3) | 54 (23.6) | <.0001 | 103 (50.5) | 72 (37.7) | .0124 | 117 (65.0) | 90 (53.3) | .0285 |
| **2 hours** | 129 (54.9) | 73 (31.9) | <.0001 | 134 (65.7) | 76 (39.8) | <.0001 | 136 (75.6) | 97 (57.4) | .0004 |
| **2.5 hours** | 131 (55.7) | 85 (37.1) | <.0001 | 135 (66.2) | 71 (37.2) | <.0001 | 133 (73.9) | 94 (55.6) | .0004 |
| **3 hours** | 139 (59.1) | 84 (36.7) | <.0001 | 139 (68.1) | 73 (38.2) | <.0001 | 126 (70.0) | 84 (49.7) | .0002 |
| **3.5 hours** | 140 (59.6) | 89 (38.9) | <.0001 | 138 (67.6) | 70 (36.6) | <.0001 | 128 (71.1) | 75 (44.4) | <.0001 |
| **4 hours** | 149 (63.4) | 86 (37.6) | <.0001 | 141 (69.1) | 67 (35.1) | <.0001 | 128 (71.1) | 74 (43.8) | <.0001 |
| **6 hours** | 149 (63.4) | 87 (38.0) | <.0001 | 134 (65.7) | 68 (35.6) | <.0001 | 122 (67.8) | 70 (41.4) | <.0001 |
| **9 hours** | 144 (61.3) | 79 (34.5) | <.0001 | 134 (65.7) | 65 (34.0) | <.0001 | 117 (65.0) | 61 (36.1) | <.0001 |
| **12 hours** | 150 (63.8) | 83 (36.2) | <.0001 | 138 (67.6) | 72 (37.7) | <.0001 | 122 (67.8) | 64 (37.9) | <.0001 |
| **24 hours** | 151 (64.3) | 81 (35.4) | <.0001 | 138 (67.6) | 68 (35.6) | <.0001 | 120 (66.7) | 61 (36.1) | <.0001 |
| **48 hours** | 145 (61.7) | 78 (34.1) | <.0001 | 129 (63.2) | 63 (33.0) | <.0001 | 110 (61.1) | 51 (30.2) | <.0001 |
|  | **Without Rescue Medication Censoring** | | | | | | | | |
|  | **Absence of Photophobia** | | | **Absence of Phonophobia** | | | **Absence of Nausea** | | |
| *Patients, n (%)* | **Eptinezumab 100 mg (n=235)** | **Placebo (n=229)** | **p-value** | **Eptinezumab 100 mg (n=204)** | **Placebo (n=191)** | **p-value** | **Eptinezumab 100 mg (n=180)** | **Placebo (n=169)** | **p-value** |
| **0.5 hours** | 33 (14.0) | 28 (12.2) | .57 | 48 (23.5) | 35 (18.3) | .22 | 66 (36.7) | 43 (25.4) | .0226 |
| **1 hour** | 72 (30.6) | 39 (17.0) | .0006 | 86 (42.2) | 52 (27.2) | .0021 | 100 (55.6) | 80 (47.3) | .11 |
| **1.5 hours** | 100 (42.6) | 56 (24.5) | <.0001 | 105 (51.5) | 73 (38.2) | .0095 | 119 (66.1) | 93 (55.0) | .0377 |
| **2 hours** | 133 (56.6) | 76 (33.2) | <.0001 | 139 (68.1) | 78 (40.8) | <.0001 | 140 (77.8) | 99 (58.6) | .0001 |
| **2.5 hours** | 143 (60.9) | 99 (43.2) | .0001 | 151 (74.0) | 89 (46.6) | <.0001 | 152 (84.4) | 118 (69.8) | .0011 |
| **3 hours** | 164 (69.8) | 125 (54.6) | .0005 | 163 (79.9) | 115 (60.2) | <.0001 | 153 (85.0) | 129 (76.3) | .0402 |
| **3.5 hours** | 174 (74.0) | 152 (66.4) | .06 | 170 (83.3) | 129 (67.5) | .0003 | 160 (88.9) | 142 (84.0) | .19 |
| **4 hours** | 185 (78.7) | 152 (66.4) | .0024 | 176 (86.3) | 138 (72.3) | .0006 | 164 (91.1) | 142 (84.0) | .0422 |
| **6 hours** | 200 (85.1) | 174 (76.0) | .0113 | 176 (86.3) | 148 (77.5) | .0275 | 164 (91.1) | 149 (88.2) | .39 |
| **9 hours** | 198 (84.3) | 179 (78.2) | .09 | 183 (89.7) | 155 (81.2) | .0213 | 164 (91.1) | 142 (84.0) | .0496 |
| **12 hours** | 207 (88.1) | 193 (84.3) | .22 | 192 (94.1) | 168 (88.0) | .0367 | 173 (96.1) | 155 (91.7) | .09 |
| **24 hours** | 213 (90.6) | 194 (84.7) | .052 | 189 (92.6) | 168 (88.0) | .13 | 169 (93.9) | 151 (89.3) | .15 |
| **48 hours** | 207 (88.1) | 198 (86.5) | .56 | 182 (89.2) | 169 (88.5) | .83 | 162 (90.0) | 151 (89.3) | .87 |

Analyses conducted in patients experiencing the corresponding symptom with their qualifying migraine.
